# Pollock avoided hydrodynamic instabilities to paint with his dripping technique

**DOI:** 10.1371/journal.pone.0223706

**Published:** 2019-10-30

**Authors:** Bernardo Palacios, Alfonso Rosario, Monica M. Wilhelmus, Sandra Zetina, Roberto Zenit

**Affiliations:** 1 Instituto de Investigaciones en Materiales, Universidad Nacional Autónoma de México, Ciudad Universitaria, Ciudad de México, México; 2 Department of Mechanical Engineering, University of California Riverside, Riverside, CA, United States of America; 3 Instituto de Investigaciones Estéticas, Universidad Nacional Autónoma de México, Ciudad Universitaria, Ciudad de México, México; 4 School of Engineering, Brown University, Providence, RI, United States of America; Max Planck Institute for Dynamics and Self-Organization, GERMANY

## Abstract

Jackson Pollock’s most celebrated abstract paintings were produced with the so-called dripping technique. By pouring liquid paint with the help of a stick or from a can, Pollock deposited viscous fluid filaments on a horizontal canvas, rhythmically moving around it. The intricate webs of lines, ubiquitous in his compositions, have fascinated art historians and scientists. Based on image analysis of historical video recordings, we experimentally reproduced the painting process. We conclude that Pollock avoided the appearance of the hydrodynamic instabilities, contrary to what was argued by previous studies. Pollock selected the physical properties of the paint to prevent filament fragmentation before deposition, and applied it while moving his hand sufficiently fast and at certain heights to avoid fluid filaments from coiling into themselves. An understanding of the physical conditions at which these patterns were created is important to further art research and it can be used as a tool in the authentication of paintings.

## Introduction

Considered one of the most prominent American painters of the 20^*th*^ century, the life and work of Jackson Pollock have been the subject of books, movies, and documentaries [1–3]. His paintings can be broadly categorized as being abstract-expressionist. Although his painting style evolved during his sometimes tormented life, the so-called ‘dripping’ technique is certainly the most widely recognized both by experts and the general public.

Jackson Pollock described the technique himself [4]. In summary, Pollock would lay a canvas horizontally and pour paint on top of it, in a controlled manner. To regulate the flow of paint, he either used an instrument (a stick, knife or a brush), poured it directly from a can and in some instances he also used a syringe. Viscous fluid filaments were produced and laid over the canvas while ‘rhythmically moving’ around it. It is believed that Pollock developed this technique strongly influenced by an experimental painting workshop, organized in New York by Mexican muralist David Alfaro Siqueiros in 1936 [5]. Interestingly, Siqueiros himself also developed the ‘accidental painting’ technique during this workshop, which was recently analyzed by Zetina *et al*. [6].

It is important to emphasize that the technique used by Pollock has been incorrectly named ‘dripping.’ The term, in the fluid mechanics’ literature, refers to the break up of a fluid jet onto drops resulting from a surface tension instability [7]. As discussed below, for the condition under which Pollock painted, the fluid filaments rarely fragmented while they were applied. Note that the formation, motion, and stability of fluid filaments have been vastly studied because of their prominence in a wide range of flow phenomena [8].

**Fig 1:**
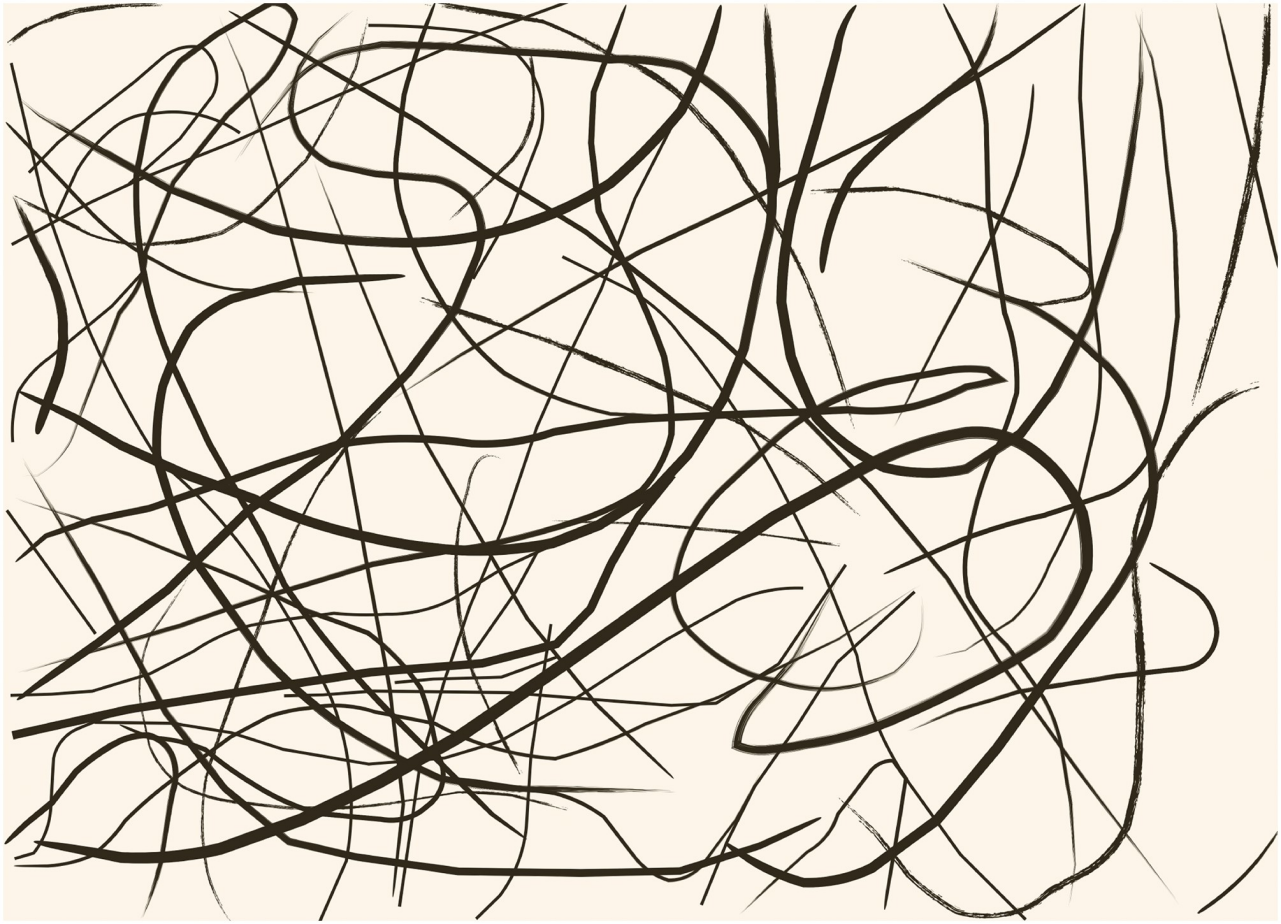
Schematic view of a small portion (lower right) of ‘Number 14: Gray’, by Jackson Pollock (1948). The image size is approximately 30×20 cm^2^. It shows only lines. The original painting can be seen in [9].

A clear illustration of the technique can be observed in ‘Number 14: Gray’ [9]. Fig 1 is a representation of a small region (lower right) of this painting, showing only lines (for the original piece refer to [9]). This particular painting is illustrative because it was painted using only one color; hence, the result of the technique is evident. It was made using black enamel paint over gesso-covered paper. Fluid filaments are organized in a characteristic manner. Although there are lines of different thicknesses and shapes, the more frequent ones are thin, unbroken and relatively straight. The curves that are observed in the fluid filaments are smooth; their radii of curvature are large. In contrast to what Herzcynski *et al*. [10] argued, here we demonstrate that the vast majority of Pollock’s traces result from decidedly avoiding the occurrence of the coiling instability [11].

### Coiling of a falling viscous filament

The phenomenon of liquid rope coiling is central to understand the dripping technique. It can be summarized as follows: the gravitational motion of a slender fluid filament falling into itself becomes unstable, under certain conditions, developing a coiling motion. Due to its relevance to many related subjects, it has been studied extensively [11–16]. In particular, the recent review by Ribe *et al*. [11] summarizes the nature of the instability and the current understanding of this process. The onset of this instability is full of subtle complexities, mainly due to the large number of parameters involved.

For the simplest case of a Newtonian filament with negligible surface tension effects, four dimensionless groups are relevant [16]:

$$\begin{array}{ccc} \Omega^{*} & = & \Omega {\left(\frac{\nu }{g^{2}}\right)}^{1/3} \end{array}$$ (1)

$$\begin{array}{ccc} H^{*} & = & H{\left(\frac{g}{\nu^{2}}\right)}^{1/3} \end{array}$$ (2)

$$\begin{array}{ccc} Q^{*} & = & Q{\left(\frac{g}{\nu^{5}}\right)}^{1/3} \end{array}$$ (3)

$$\begin{array}{ccc} d^{*} & = & d{\left(\frac{g}{\nu Q}\right)}^{1/4} \end{array}$$ (4)

where Ω is the coiling frequency, *ν* is the fluid kinematic viscosity, *g* is the gravitational acceleration, *H* is the height from which the fluid is dispensed, *Q* is the fluid volumetric flow rate, and *d* is the filament diameter at the height *H*. Hence, the following functional relation can be expected:

$$\begin{array}{c} \Omega^{*}=\Phi \left(H^{*},Q^{*},d^{*}\right). \end{array}$$ (5)

In other words, to know the value of Ω*, the height, flow rate, and filament diameter must be known, in addition to the fluid viscosity and gravitational acceleration.

In most experiments, both the flow rate and the diameter of the filament are kept fixed; hence, it is possible to draw a plot of coiling frequency, Ω*, as a function of height, H* (see for instance Fig 5 in [11]). For this case, as the value of H* increases, several regimes are observed: viscous, gravitational, inertio-gravitational, and inertial. In general, however, Ω* depends also on Q* and d*. Therefore, the relation in Eq 5 must be known.

Furthermore, if the surface tension effect is relevant and is also included in the analysis, an additional dimensionless group emerges. One reasonable option is to consider the Ohnesorge number:

$$\begin{array}{c} Oh=\nu \sqrt{\frac{\rho }{\sigma d}} \end{array}$$ (6)

where *ρ* is the fluid density and *σ* is the surface tension. If the value of *Oh* is large, surface tension effects can be considered negligible. The value of the Ohnesorrge number also determines whether or not a long viscous filament will fragment or not, as discussed by [17]. Note, however, that in the present case gravitational effects are important. Therefore, it is relevant to compare the surface tension effects with gravitational stretching:

$$\begin{array}{c} \Pi_{\sigma }=\frac{\rho gHd}{\sigma }. \end{array}$$ (7)

If both *Oh* > 1 and Π_*σ*_ > 1, then surface tension effects can be considered negligible.

Another property which can be relevant for the case of paints is the viscoelasticity of the liquid [18]. If the fluid is viscoelastic, characterized by a relaxation time λ, another dimensionless group ought to be considered. The Deborah number is defined as:

$$\begin{array}{c} De=\lambda \frac{Q}{d^{2}H}. \end{array}$$ (8)

If *De* ≪ 1, the viscoelastic effect can be discarded. Furthermore, the viscoelastic relaxation time can also be compared to the characteristic gravitational time leading to:

$$\begin{array}{c} \Pi_{\lambda }=\lambda \sqrt{\frac{g}{H}}. \end{array}$$ (9)

For viscoelastic effects to be considered unimportant both Π_λ_ and *De* would have to be small.

### Fluid mechanical sewing machine

In the context of the present study, it is important to consider the case when the substrate and the nozzle issuing the fluid have a relative velocity. Chiu-Webster and Lister [19], for example, studied the case for which the displacement of a moving substrate stretched a coiling fluid filament. They coined the term ‘fluid mechanical sewing machine,’ because the forming coils move sideways as they are deposited on the moving substrate, resembling sewing patterns. Several shape regimes were observed and analyzed. This phenomena has since then been studied extensively [12–15] and is very well understood. In particular, the case in which the coiling instability can be entirely prevented: if the relative speed between substrate and nozzle is sufficiently large, the fluid filament is deposited on the substrate in a straight line, without curls. Ribe *et al*. [20] evaluated the conditions needed to prevent coiling resulting from the motion of the substrate. This case will be analyzed below.

## Materials and methods

### Image processing of historical videos

We conducted a series of measurements of the speed and height at which Pollock painted. These measurements were obtained from the documentary video from Hans Namuth [21], in which Pollock was filmed while painting. The measurements were conducted by using the free software Tracker. In essence, we measured distances on the screen over time. To obtain physical dimensions, we assumed that the size of Pollock’s hand was *H*_*hand*_ = 20 cm.

**Fig 2:**
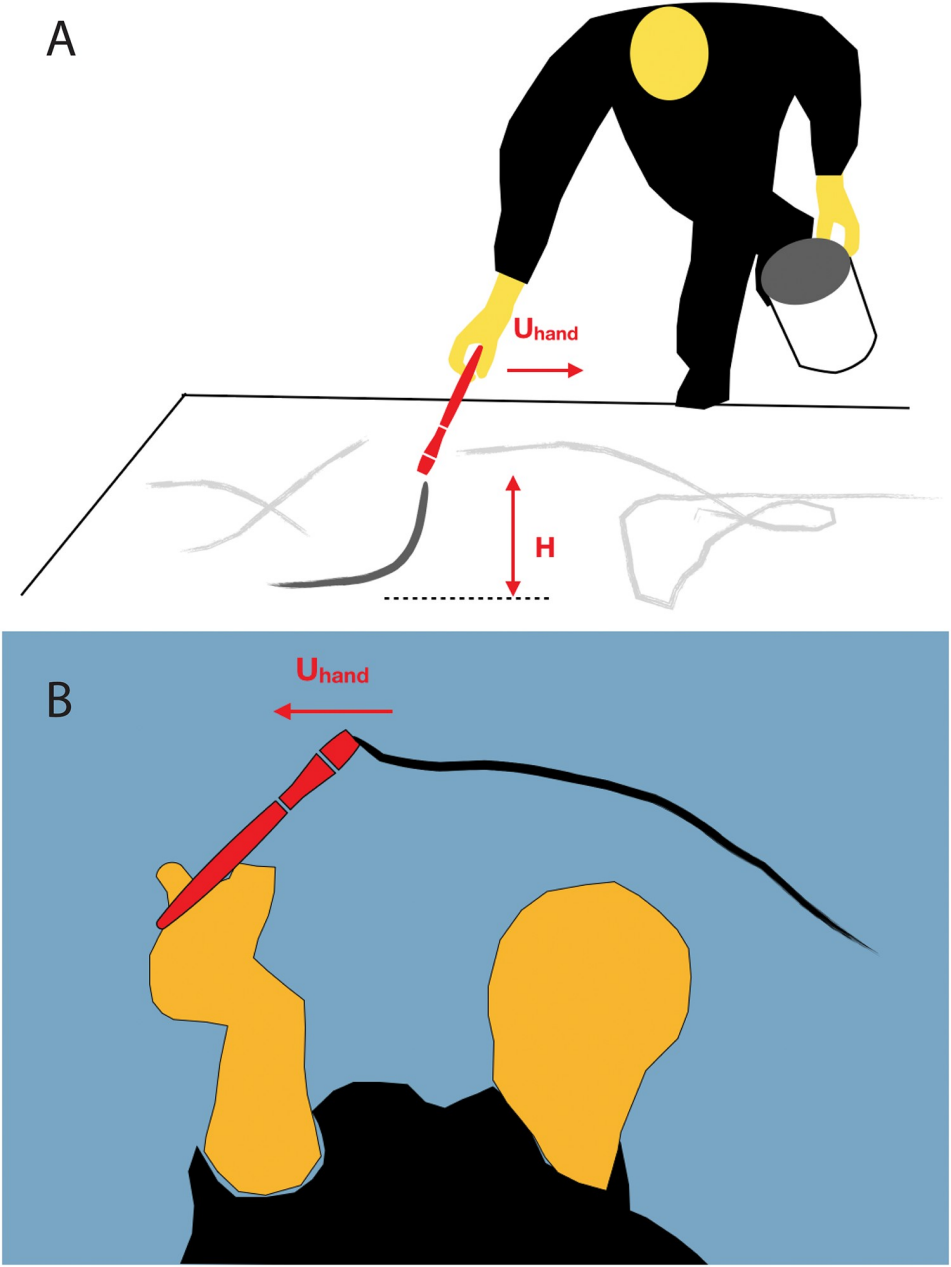
Illustration of Pollock’s painting action. (A) Side view of Pollock painting, while moving around the canvas. The two quantities measured from the videos, *U*_*hand*_ and *H*, are shown; (B) Pollock painting over a transparent glass sheet. The drawings are schematic reproductions of Hans Namuth’s documentary [21].

Fig 2A shows a schematic view of Pollock during the painting process, reproduced from a snapshot of the movie. From the image sequences, three quantities were measured: the speed at which the hand moves, *U*_*hand*_, the height from which the paint drips, *H*, depicted in Fig 2A, and the loading speed, *U*_*load*_ (the speed at which the stick is retrieved from the can of paint). *U*_*load*_ is used to determine the amount of fluid and the flow rate dripping from the stick.

We analyzed side-view sequences for which the distance to the canvas could be measured in time. In parts of the movie, Pollock painted on top of a transparent glass sheet, as shown schematically in Fig 2B. These images allowed us to measure the hand speed. A large sample of speeds and heights were collected to obtain statistical distributions, from which the mean and standard deviation were calculated. The height was measured from side images, as shown in the illustration of Fig 2A. Due to the low resolution of the video, the uncertainty of these measurements is within 26%. The raw data from these measurements has been included as Supplementary Material.

### Experimental setup

**Fig 3:**
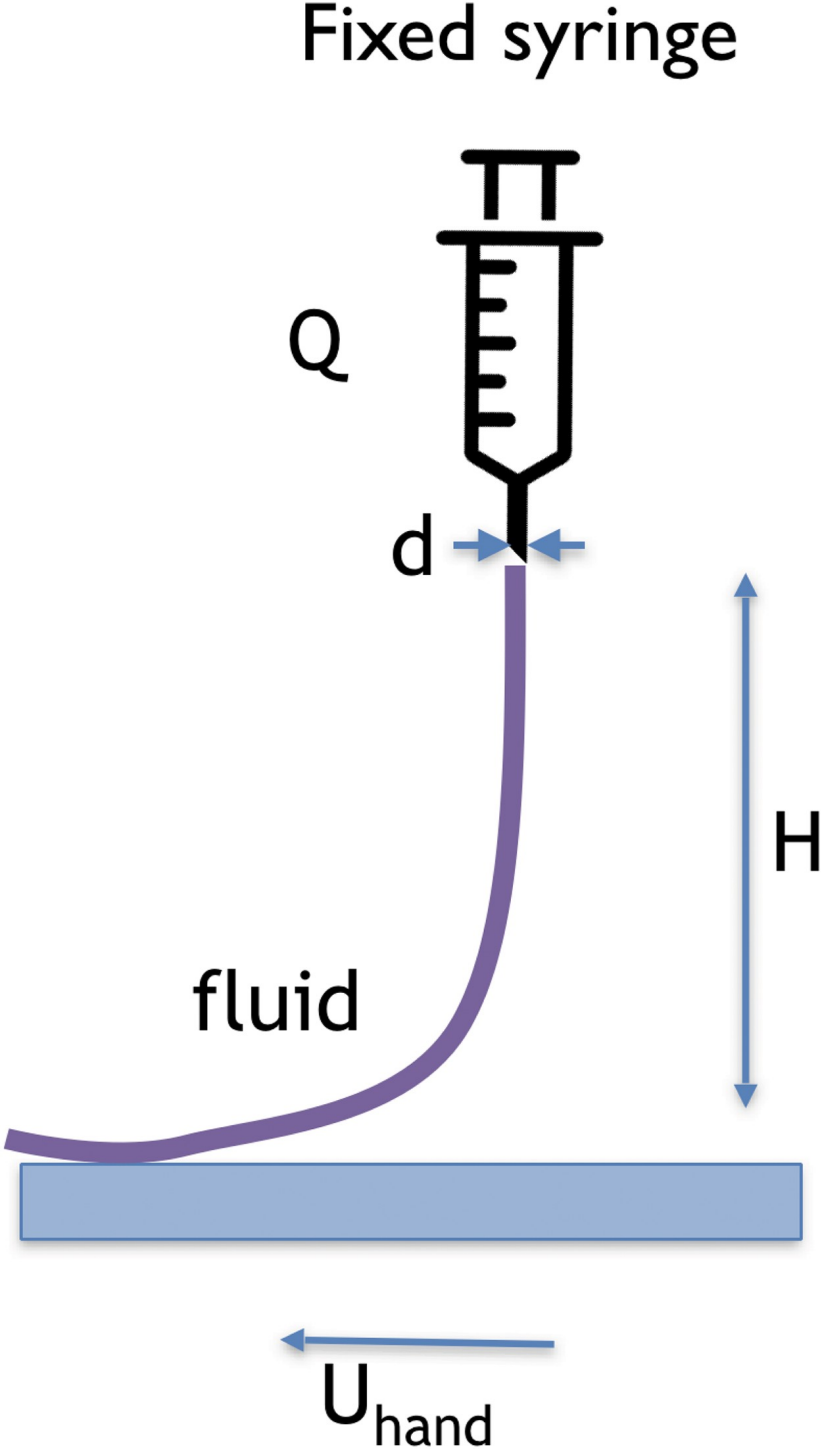
Schematic view of the experimental setup.

To reproduce the technique, we used an experimental setup similar to that of [12–15, 19], shown schematically in Fig 3. It consists of a stand that holds a syringe, aligned vertically, at a certain height, *H*, above the horizontal substrate onto which the paint is dripped. The syringe is mounted on a syringe pump such that it produces a constant flow rate, *Q*. The syringe exit nozzle has a diameter *d*. As the fluid leaves the syringe, it forms a continuous steady filament that thins as it falls under gravity, reaching a diameter *d*_*o*_ as it reaches the substrate. The mean fluid velocity leaving the nozzle, *U*, can be calculated as *U* = 4 *Q*/(*πd*^2^). To recreate the motion of the hand (as done by the artist and depicted in Fig 2A), we chose to move the substrate and keep the syringe nozzle fixed. The substrate, which is thick white paper, moves at a constant speed, *U*_*hand*_. Its motion is achieved by a pair of rubber wheels that are rotated at a constant rate by a step motor. By setting the rotation rate of the wheels, different values of *U*_*hand*_ can be achieved. Note that changing the configuration, by keeping the *hand* fixed and moving the substrate, implies a different set of boundary conditions that may affect the onset of the coiling instability. We neglect this effect.

### Test fluids

We chose to use a paint similar to that used by Pollock in some of his works. The paint is a commercial black cellulose Nitrate lacquer. Most likely, the paints used by Pollock had a different composition, leading to different values of viscosity and density. We take this value as our starting point; we discuss the effect of either increasing or decreasing fluid viscosity in the formation of filaments. To extend the parameter range, the lacquer was diluted with commercial thinner to reduce its viscosity. The fluid density remains practically unchanged.

Table 1 shows the properties of the paint and its dilutions. The rheological properties were obtained using a plate-plate rheometer (Anton Paar, Physica MCR101). The density was measured with a 25 ml pycnometer. The value of the surface tension was not measured, but rather inferred from [22] (a value of *σ* = 40 mN/m was considered for all cases). The paint was nearly Newtonian; it had nearly constant viscosity, *μ*_*o*_, for very low shear rates. Its shear-thinning nature, quantified by the power index, *n*, is small (*n* ≈ 1). To assess the viscoelastic nature of the fluid, oscillatory rheological tests were conducted. Considering the technique used by [23], we fitted the curves of the storage and loss moduli to a generalized Maxwell model to obtain a mean value of the relaxation time, *τ*, which was found to be of O(10^−3^) s, indicating weak viscoelastic effects for low shear rates.

**Table 1:** Physical properties of paints used in the study.

Paint	Density *ρ*, kg/m^3^	Viscosity *ν*, m^2^/s	Power Index *n*, -	Relaxation Time λ, s	Flow Rate *mm*^3^/*s*
Black, B1, (○,●)	1002	4.99×10^−3^	0.85	1.9×10^−3^	162
Black, B2, (Δ,▲)	995	2.81×10^−3^	0.85	1.7×10^−3^	220
Black, B3, (▽,▼)	995	4.00×10^−3^	0.85	1.8×10^−3^	220
Black, B4, (□,■)	997	3.2×10^−3^	0.85	1.8×10^−3^	220
Black, B5, (◇,◆)	997	3.0×10^−3^	0.85	1.8×10^−3^	220

An experimental campaign was conducted for each fluid, varying the height, *H*, and the speed of the substrate, *U*_*hand*_. Hence, for each campaign, the values of the nozzle diameter, flow rate, and fluid properties were kept fixed. The nozzle diameter was *d* = 2.0 mm for all cases, while the flow rate varied slightly for each case (Table 1). The range of heights explored in the study was 10 < *H* < 120 mm; the speed of substrate was 25 < *U*_*hand*_ < 1000 mm/s.

The values of the relevant dimensionless numbers for the five experimental campaigns conducted are shown in Table 2, considering the physical properties of the fluids shown in Table 1 of the main text. From maximum values of *De* and Π_λ_, we can conclude that viscoelastic effects are small. Considering the minimum values of *Oh* and Π_*σ*_, it is clear that surface tension effects are unimportant for the current experiments.

**Table 2:** Values of the relevant dimensionless numbers of the experiments conducted in this study. For the groups *Oh* and Π_*σ*_, only the minimum values are reported; for *De* and Π_λ_, only maximum values are shown.

Fluid	H*	Q*	d*	*Oh*	Π_*σ*_	*De*	Π_λ_
Black, B1, (○,●)	0.73–7.32	2.4×10^−3^	6.6×10^−1^	17.7	4.9	6.7×10^−4^	1.8×10^−2^
Black, B2, (Δ,▲)	6.44–14.44	8.4×10^−3^	7.1×10^−1^	9.9	4.9	9.3×10^−3^	5.3×10^−2^
Black, B3, (▽,▼)	5.91–10.22	4.7×10^−3^	6.5×10^−1^	14.1	4.9	9.9×10^−3^	5.6×10^−2^
Black, B4, (□,■)	9.87–11.85	6.8×10^−3^	6.9×10^−1^	11.3	4.9	9.9×10^−3^	5.6×10^−2^
Black, B5, (◇,◆)	6.18–11.34	7.6×10^−3^	6.9×10^−1^	10.6	4.1	5.5×10^−3^	3.1×10^−2^

### Measurement of the flow rate dripping from a stick

As explained above, Pollock frequently used sticks to withdraw paint from the can. After immersing the stick in the can, the can was drawn upwards to capture a certain amount of fluid. The loaded stick was then placed over the horizontal canvas and displaced at a certain speed and height over it. The amount of fluid issuing from the stick, *Q*_*stick*_, needs to be known to determine if the filaments placed on the horizontal canvas would coil or not.

The amount of fluid drawn by the stick during its vertical motion can be calculated from the classical Landau-Levich problem [24]; however, in the present case, the surface tension effects are small. Hence, the calculation from the Landau-Levich problem would not be applicable to the set of conditions relevant to our study. Herczynski *et al*. [10] proposed a viscous scaling for *Q*_*stick*_ leading to

$$\begin{array}{c} Q_{stick}∼D_{stick}\sqrt{\frac{\nu }{g}}U_{load}^{3/2}\;, \end{array}$$ (10)

where *U*_*load*_ is the speed at which the stick is withdrawn from the fluid bath and *D*_*stick*_ is the diameter of the stick.

**Fig 4:**
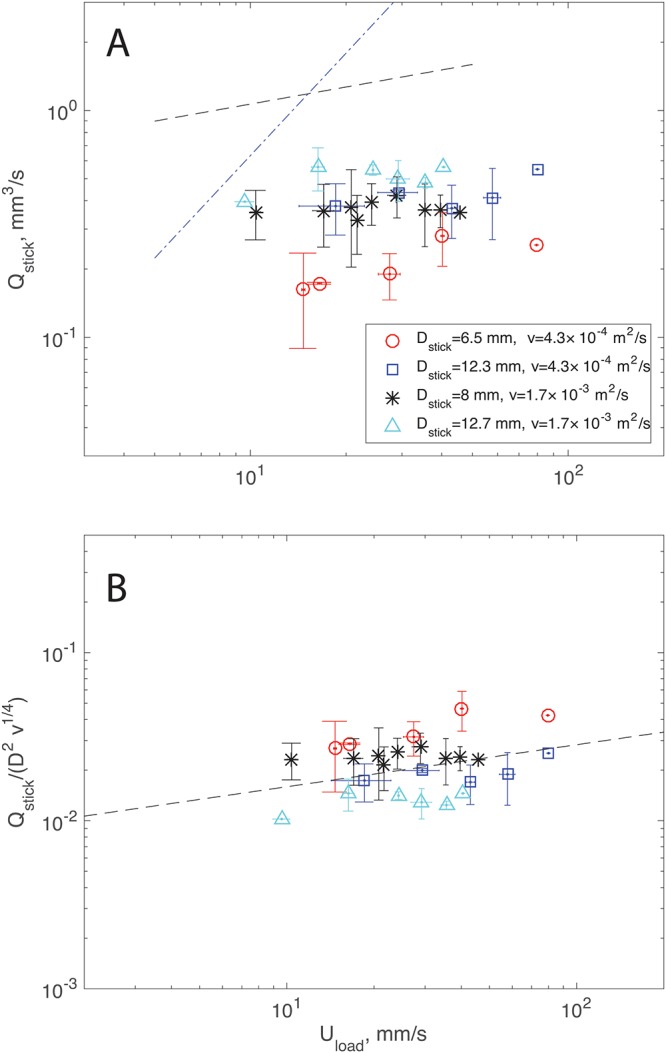
(A) Stick flow rate, *Q*_*stick*_, as a function of loading speed, *U*_*load*_, for three different sticks and two fluids; the black dashed line shows a trend of $Q_{stick}∼U_{load}^{1/4}$, while the blue dashed-dotted line shows the trend $Q_{stick}∼U_{load}^{3/2}$. (B) Ratio $Q_{stick}/\left(D_{stick}^{2}\nu^{1/4}\right)$ as a function of loading speed, *U*_*load*_; the prediction of Eq 11 is shown by the dashed-dotted line.

To validate Eq 10, we built an additional experimental setup to reproduce the idea depicted in Fig 4 of [10]. The flow rate was measured for three different stick diameters, two fluid viscosities, and a range of loading speeds. The experimental results are shown in Fig 4A in dimensional terms. As expected, the flow rate increases with loading speed but not at the rate of $U_{load}^{3/2}$ predicted by Eq 10 (depicted by the dashed-dotted line). The flow rate, instead, appears to increase as $U_{load}^{1/4}$ (dashed line) for all the cases tested. The flow rate also appears to increase with fluid viscosity, *ν*, and stick diameter, *D*_*stick*_.

The results scale as:

$$\begin{array}{c} Q_{stick}=\kappa D_{stick}^{2}{\left(g\;\nu \;U_{load}\right)}^{1/4}\;, \end{array}$$ (11)

where *κ* = 0.016. Note that we do not have a physical interpretation for this equation, but its dimensional homogeneity was ensured. Despite the scatter in the data, the fitting is satisfactory. Therefore, we use Eq 11 to determine the flow rate issuing from a stick that, in turn, can be used to estimate the flow rate used by Pollock. Also note that the discussion above does not consider the fact that the flow rate is expected to decrease in time, as the paint drips off the stick.

## Results

### The painting action

**Fig 5:**
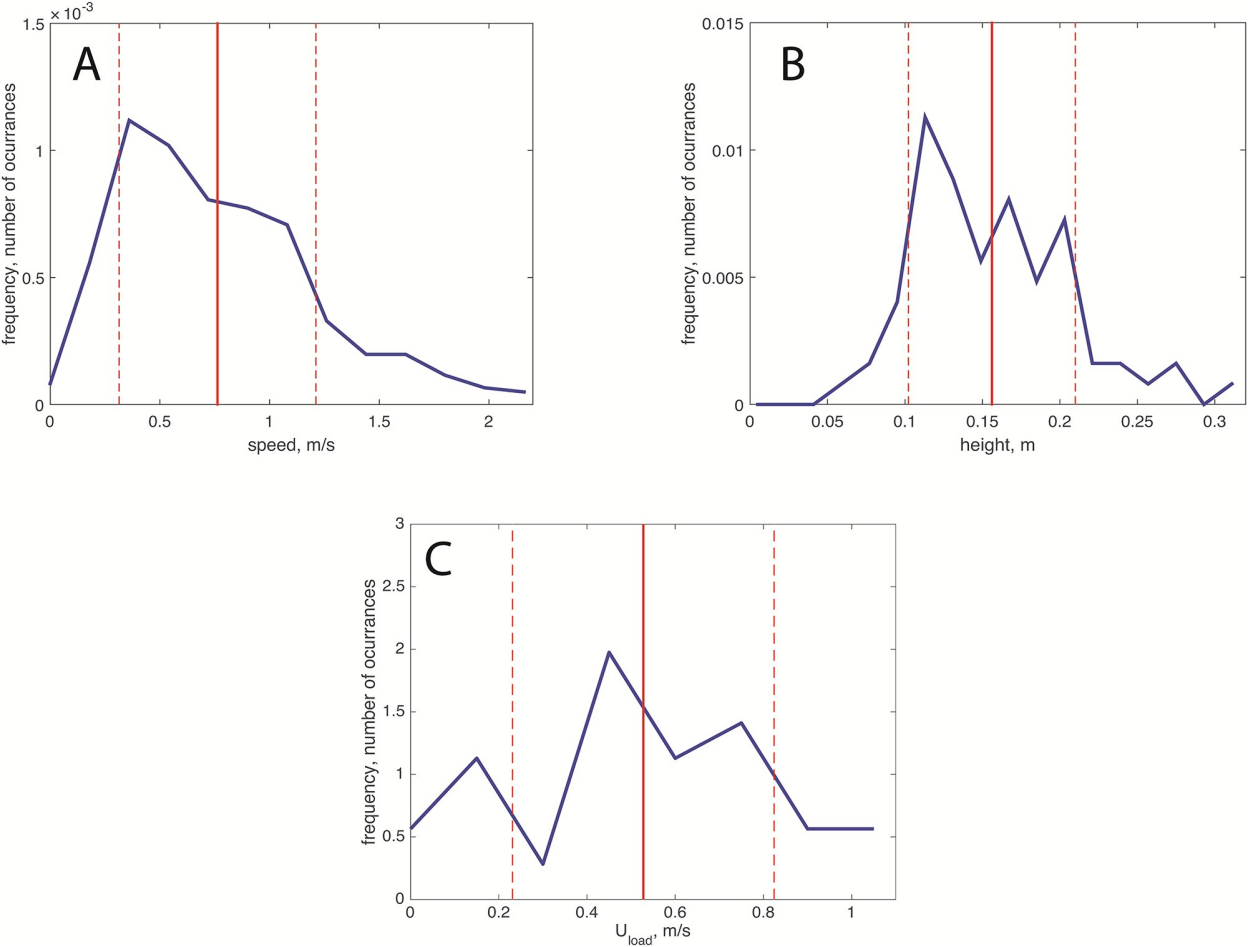
Statistics of Pollock’s painting action. (A) Histogram of the hand speed, *U*_*hand*_; (B) histogram of the height of the hand from the surface of the canvas, *H*; histogram of the loading speed, *U*_*load*_. The histograms are normalized such that the integral over the frequencies is unity. The vertical solid lines show the mean values in each case $\bar{x}$*x*. The vertical dashed lines to the right and left of $\bar{x}$ represent $\bar{x}+\sigma_{x}$ and $\bar{x}-\sigma_{x}$, respectively, where *σ*_*x*_ is the standard deviation.

To understand the fluid mechanics of Pollock’s technique, measurements of the painting action were obtained from historical videos (described in detail above). The histogram of the hand speed is presented in Fig 5A. In the plot, the vertical line shows the mean value; the dashed lines show the standard deviation. The distribution of speeds is not symmetric with respect to the mean value, being positively skewed. This log-normal-like distribution indicates that the hand movements are not arbitrary but rather conducted in a calculated manner. The histogram of heights, shown in Fig 5B, is also positively skewed, but the range of values around the mean are more uniformly distributed. Fig 5C shows the distribution of *U*_*load*_. The mean loading speed was *U*_*load*_ = 52.8 cm/s with a large standard deviation of 29.7 cm/s. Note that these speeds are used to calculate *Q*_*stick*_ used by Pollock.

Due to the low resolution of the videos, we were not able to obtain simultaneous height and hand speed measurements. Therefore, it was not possible to correlate these two quantities.

It is also relevant to note that, from the historical videos, the stick used to manipulate the fluid was not held vertically. During the application of the paint to the canvas, the stick would be swung from roughly -30° to +30° (0° corresponding to vertical orientation). Since, at a maximum inclination, the flow rate is expected to decrease by 13%, as only *g* cos 30° would drive the downward flow, the additional effect due to the inclination of the stick is not substantial and was neglected in the analysis.

### Fragmentation?

From Pollock’s paintings, like that in [9], we determined that the typical thickness of the filaments is *d*_1_ ≈ 3 mm. Comparing this thickness to the mean height from which the filament is being deposited, we calculate their aspect ratio, Γ = *H*/*d*_1_. This metric indicates the slenderness of the fluid filaments, which can be used to assess if they will fragment or not. From the measurement of the mean height, we estimate Γ_*Pollock*_ ≈ 50. According to Driessen *et al*. [17], a viscous filament with an aspect ratio Γ < *Oh*^2^ would remain stable, where *Oh* is the Ohnesorge number (defined by Eq 6).

For the case of Pollock’s paintings, considering the physical properties of paint B1 (see Table 1), and a typical value of *d* = 2 mm, would lead to *Oh*_*Pollock*_ ≈ 18. Therefore the condition on the slenderness for the filaments to fragment would be Γ_*Pollock*_ = 324, which is significantly larger than the critical fragmentation value [17]. Therefore, the viscous filaments used by Pollock would remain stable and would not fragment into droplets. It is important to mention that the expression Γ < *Oh*^2^ from [17], valid for very viscous liquids, underestimates the stability of a filament because it does not account for gravitational stretching, which is known to increase filament stability [8]. Furthermore, note that (Π_*σ*_)_*Pollock*_ ≈ 120, indicating that gravitational forces are much larger than surface tension ones, which confirms the fact that fragmentation of fluid filaments rarely occurred in Pollock’s traces.

Also, from the video measurements, considering the mean values we calculate (*De*)_*Pollock*_ ≈ 1.2 × 10^−4^ and (Π_λ_)_*Pollock*_ ≈ 7.9 × 10^−3^, which indicates that viscoelastic effects were not relevant for Pollock’s painting action.

### Coiling?

To determine if the traces produced by Pollock are expected to coil or not, we first need to identify the range of conditions under which the paint was deposited. From the results shown in Fig 5C, we know that 0.05 < *H* < 0.25 m. Considering the properties of black paint (fluid B1, Table 1), these heights can be recast in dimensionless terms considering Eq 2, leading to 2.5 < H* < 12.2. The expected flow rate can be determined using Eq 11, considering *D*_*stick*_ = 0.5 cm, *U*_*load*_ = 52.8 cm/s (from Fig 5C), and again the properties of fluid B1. In dimensionless terms, using Eq 3, we obtain $Q_{stick}^{*}=2.5\times 10^{-3}$. Also, by assuming a filament diameter of *d* = 3 mm, and the same fluid properties, we obtain from Eq 4, that d* = 0.98. Under these conditions, according to [16], a non-moving filament would coil. Note also that under these conditions *gQ*^2^*H*/*ν*^4^ = 7.2 × 10^−5^, which is below the threshold value for the cessation of coiling proposed by [20]. According to Ribe *et al*. [11], for this range of heights, the coiling would occur within the inertial regime.

As discussed by Herczynsky *et al*. [10], the coiling instability can be prevented if the displacement velocity is as large as the curling speed. Therefore, the transition from coiling to straight lines is expected to occur if:

$$\begin{array}{c} \frac{U_{hand}}{\Omega R}\geq 1, \end{array}$$ (12)

where Ω and *R* are frequency and radius of coiling, respectively. Since both Ω and *R* change with height, different transition conditions are to be expected depending on the coiling regimes.

For the inertial regime, according to [25], the coiling frequency scales as:

$$\begin{array}{c} \Omega ∼{\left(\frac{Q^{4}}{\nu d_{1}^{10}}\right)}^{1/3}, \end{array}$$ (13)

where *Q* is the volumetric flow rate of fluid; *d*_1_ is the filament diameter as it reaches the ground, which scales as:

$$\begin{array}{c} d_{1}∼{\left(Q^{2}/gH\right)}^{1/4}. \end{array}$$ (14)

Ribe [16] found that, for a filament with negligible viscous resistance to stretching, the coiling frequency can be re-written in more familiar terms as:

$$\begin{array}{c} \Omega ∼\frac{Hg}{\nu }. \end{array}$$ (15)

Assuming that *R* ∼ *d*_1_, we can write:

$$\begin{array}{c} \Omega R∼{\left(H^{*}\right)}^{3/4}{\left(Q^{*}\right)}^{1/2}{\left(g\nu \right)}^{1/3}. \end{array}$$ (16)

It is important to emphasize that we are assuming that the coiling frequency and radius are those at the onset of coiling and that they are not affected by the fact that the filament is moving with respect to the substrate. Since the tension in the filament would certainly change with fluid displacement, we expect both Ω and *R* to change. In this analysis, we assume that such change is negligible.

Therefore, the critical condition for transition can be expressed in terms of other physical variables of the problem:

$$\begin{array}{c} \frac{U_{hand}}{\Omega R}∼\frac{U^{*}}{{\left(Q^{*}\right)}^{1/2}{\left(H^{*}\right)}^{3/4}}, \end{array}$$ (17)

where U* = *U*_*hand*_/(*gν*)^1/3^ is a normalized speed and Q* = *Q*(*g*/*ν*^5^)^1/3^ is the normalized flow rate. Therefore, the transition from curled to straight lines would be expected when:

$$\begin{array}{c} U^{*}\;≳\;{\left(Q^{*}\right)}^{1/2}{\left(H^{*}\right)}^{3/4}. \end{array}$$ (18)

Now, to obtain the proportionality constant in Eq 18, experiments were conducted. The setup is described in detail in the Methods Section. In essence, fluid filaments were generated with a syringe, elevated at a height *H*, above a horizontal substrate that moved at a constant speed *U*_*hand*_. The setup emulates Pollock’s painting action, with the difference that in this case the ‘hand’ is fixed and the substrate steadily translates. Experiments were conducted for several fluids, and for a range of heights and speeds to produce traces which were either straight or coiled.

**Fig 6:**
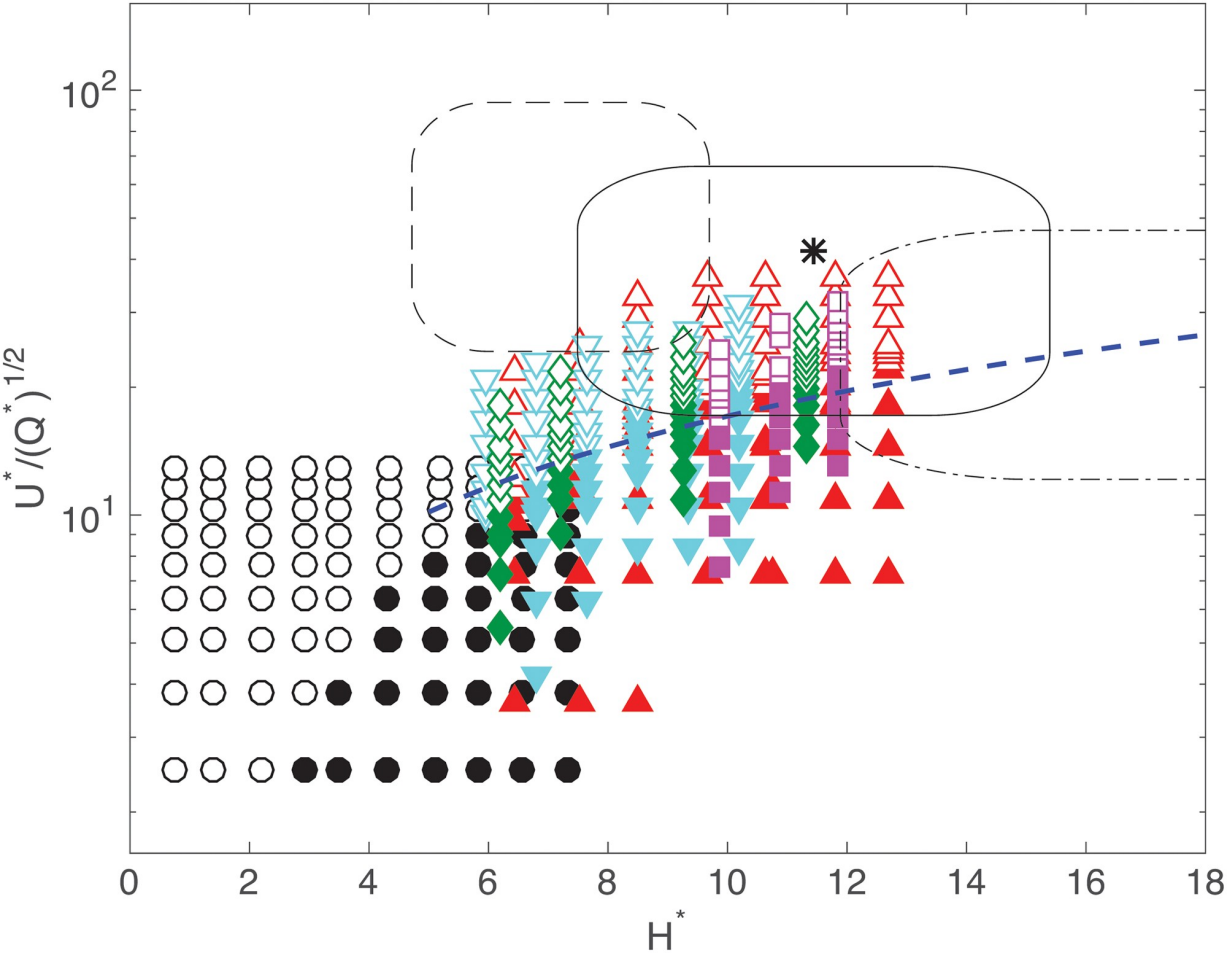
Map of the ratio $U^{*}/\sqrt{Q^{*}}$, as function of normalized height, H*. The empty and filled symbols correspond to straight and curled traces, respectively. Each symbol represents experiments for different fluids, see Table 1. The black asterisk shows ($H_{Pollock}^{*},{\left(U^{*}/\sqrt{Q^{*}}\right)}_{Pollock})$, considering the measurements shown in Fig 5, the physical properties of black paint B1 (from Table 1) and the calculation of $Q_{stick}^{*}$ (from Eq 11). The solid-line rectangle shows a region considering one standard deviation (plus and minus) around mean values of height and speed. The dashed and dashed-dotted rectangles show the regions that correspond to fluids with twice and half the value of the viscosity of fluid B1, respectively. The thick dashed line shows the prediction from Eq 19.

Fig 6 shows the experimental measurements, separated into two groups (straight and curled traces). For a given height, the traces are curled for small velocities; as the speed increases, a transition into straight lines is observed. As the height increases, the speed at which the transition occurs is larger. For H* > 8, a line is fitted through the transition considering the trend of Eq 18:

$$\begin{array}{c} U^{*}=\kappa {\left(Q^{*}\right)}^{1/2}{\left(H^{*}\right)}^{3/4}, \end{array}$$ (19)

for which *κ* = 3.04 fits the transition relatively well.

Another, more direct, manner to assess the cessation of coiling resulting from the relative displacement from the hand and ground can be formulated considering the condition

$$\begin{array}{c} \frac{U_{hand}}{U_{f}}\geq 1, \end{array}$$ (20)

where *U*_*f*_ is the speed of the flow when it reaches the ground. Interestingly, Ribe *et al*. [20] demonstrated that *ΩR* and *U*_*f*_ scale in the same manner. Hence, the two criteria would lead to similar transition conditions.

## Discussion

Using the mean height and speed measurements from historical videos, Pollock’s painting action is located on the map shown in Fig 6 to determine whether the parameter space corresponds to regions where coiling instabilities are expected. The black asterisk in the figure is estimated from the mean values of his painting action (from Fig 5C and 5D) in normalized form. The height is calculated as $H_{Pollock}^{*}=H_{Pollock}{\left(g/\nu^{2}\right)}^{1/3}$. To calculate the ratio ${\left(U^{*}/\sqrt{Q^{*}}\right)}_{Pollock}$, both the fluid properties and the value of $Q_{Pollock}^{*}$ need to be determined. To evaluate $Q_{Pollock}^{*}$, an additional experiment was conducted (see Materials and methods): a stick (a cylinder) was partially immersed in a viscous fluid and quickly drawn out as the dripping rate was measured to determine *Q*_*stick*_. The experimental results were fitted to a scaling (Eq 11) from which $Q_{stick}^{*}=Q_{stick}{\left(g/\nu^{5}\right)}^{1/3}$ can be obtained. Considering values for *D*_*stick*_ = 0.5 cm, *U*_*load*_ = 52.8 cm/s, and the properties of fluid B1 (Table 1, Methods and materials), we obtain $Q_{stick}^{*}=Q_{Pollock}^{*}=2.5\times 10^{-3}$. With this value, H* and $U^{*}/\sqrt{Q^{*}}$ for Pollock’s painting action are located in Fig 6. Additionally, a rounded box (continuous line) is drawn around the mean conditions (asterisk), whereby the width and height of the box correspond to normalized standard deviation values of speed and height. In other words, the area within the box corresponds to the range of conditions used by Pollock to paint. Most importantly, the area of the box is mostly above the transition to straight lines, within the inertial coiling regime. Therefore, we can conclude that the majority of traces drawn under these conditions would be straight lines. This conclusion is in agreement with the visual inspection of the image in Fig 1. Note that, by analyzing other pieces of Pollock where the dripping technique was used (not shown), a small fraction of coiled filaments can be readily identified. This is, again, in agreement with the results in Fig 6. Note also that a smaller value of Q*, resulting from an inclined stick or the decrease of flow in time, would move the region of painting vertically up into the straight-line region.

Since the actual properties of Pollock’s paints are not known, we can recalculate the region of painting action considering variations in the viscosity of the paints. Note that the change in viscosity changes the normalization of *U*_*hand*_ and *H*, but also the prediction of *Q*_*Pollock*_. The drawn boxes with dashed and dashed-dotted lines in Fig 6 correspond to paints with twice and half the values of the viscosity of the black paint, respectively. If the viscosity is larger, the normalized height decreases while the normalized speed increases; these changes make the entire area of the box to be well within the straight-line region. In turn, for a smaller value of the viscosity, the normalized height increases but the speed decreases slightly, making the box to be right in between the two regions (sectioned by the transition criteria). In this case, as many traces would coil or be straight; however, for this viscosity, the Ohnesorge number would also significantly decrease (i.e., *Oh* ≈ 7.5), leading to Γ = 56.3 which is very close to the fragmentation onset. One would expect some of of the fluid filaments to lose their stability and fragment. In fact, a closer inspection of Pollock’s works in the dripping period also reveals the occasional occurrence of drops (fragmented filaments). Notwithstanding the precise values of the properties of the paint, for the measured values of speed and height, the large majority of the traces would be straight lines. If we could argue that Pollock always painted with the same action (as that in videos), we can conclude that very few traces would coil. In other words, the painting action was conducted such that the appearance of the coiling instability would rarely appear.

Painters, including Pollock, commonly manipulate the fluid properties by adding solvents. Although changes in surface tension and density do occur, the viscosity is significantly affected by the addition of such additives. In general, the addition of fluid-thinners, leads to a reduction of viscosity. Considering the map shown in Fig 6, if the viscosity is smaller, the value of U* would be larger. Therefore, with less viscous fluids, coiling traces could be produced without having to move the hand too quickly. A slowly moving hand would then produce more uniform lines. Also, if the viscosity is reduced, the value of H* would increase. The filaments would thus have to be deposited from lower heights to prevent the formation of curled lines. These arguments are in good agreement with the way in which Pollock painted, as per the recordings and the measurements in Fig 5.

## Conclusions

By locating the painting action in the general map of behavior, in the appropriate dimensionless form, we demonstrate that Pollock aimed to prevent the coiling instability. This result could be of importance for authentication: a painting with too many coiled traces would indicate that the painting was not created by Pollock. Furthermore, understanding the conditions for which the coiling instability can be prevented could have implications in practical applications where such an effect needs to be prevented, as in the case of ink-jet printing or the fabrication of optic fibers.

## Supporting information

S1 File Raw data obtained from the measurements of Pollock’s painting action from the historical videos. (TXT)
