# Supplementary material for: Pollock avoided hydrodynamic instabilities to paint with his dripping technique
Source: PLoS One. 2019 Oct 30;14(10):e0223706. doi: 10.1371/journal.pone.0223706 (PMC6821064; doi:10.1371/journal.pone.0223706)
Supplement: S1 File — (TXT) [file pone.0223706.s001.txt]

%%%% Supplementary information%Raw data obtained from Hans Namuth’s documentary of Pollock’s painting technique:% Hand speed, in m/sData1=[    1.1799    0.6720    1.1291    1.7840    1.1489    0.4029    0.0915    0.2807    0.0404    0.1875    0.3368    0.3690    0.0473    0.1040    0.2562    0.3550    0.2927    0.4681    0.6177    0.6866    0.9771    0.4527    0.3490    0.5777    0.5181    0.2532    0.2137    0.1802    0.2774    0.1282    0.3214    0.1642    0.2288    0.2392    0.2334    0.2291    0.1247    0.1801    0.0678    0.2987    0.2906    0.0647    0.0693    0.2324    0.1393    0.3023    0.4617    1.8433    1.0109    0.8312    1.7093    1.0972    1.1699    1.4132    1.0719    1.2390    0.6360    0.4053    0.9150    0.7611    0.8305    0.5574    0.4632    0.4064    0.4607    0.3726    0.5016    0.2921    0.4153    0.5221    0.4855    0.4124    0.2858    0.5456    0.7779    0.4517    0.3592    0.2892    0.3133    0.1457    0.5936    0.5570    0.3901    0.3789    0.5791    0.2929    0.2639    0.2208    0.2233    0.4316    0.2999    0.6254    0.4489    1.1485    1.1491    1.1018    2.2439    1.2552    0.8334    2.3260    1.9215    2.0247    1.3891    0.5704    0.9191    0.7242    0.6988    1.0362    0.7491    0.6809    0.9090    0.7623    1.1338    1.1833    0.9947    1.1672    0.9641    0.5584    1.0512    1.5954    1.6300    0.6439    0.1572    0.5781    0.7660    1.0524    1.1360    0.5869    0.3419    0.4457    0.3789    0.4484    0.3477    0.4115    0.4913    0.6066    1.5384    1.5090    0.6403    0.7604    1.1533    1.2698    1.3768    1.7487    1.1707    0.7530    1.6910    1.5261    1.2699    1.0870    0.6706    1.0933    1.5284    0.7185    1.5371    0.8526    0.9012    0.9655    0.6018    0.9403    0.6169    0.2541    1.2611    1.1546    0.5666    0.3825    1.3386    0.9460    0.8644    0.7344    0.6847    1.1688    0.8653    0.9278    1.7927    1.1409    0.8148    1.7451    1.8833    2.2842    1.6697    0.9740    1.1444    0.8166    0.7628    0.4922    0.2390    0.4002    0.3759    0.6223    0.2330    0.5384    0.6230    0.9029    1.1193    0.7285    0.7225    1.0636    1.3518    0.7020    0.4870    1.3095    1.1643    1.0080    0.9646    1.7617    1.6160    1.9890    2.0201    1.3349    1.5591    1.3448    0.6734    0.8589    0.5494    0.4134    0.8330    0.9208    0.8833    1.1852    0.7661    0.6906    0.8446    0.8693    1.1382    1.0361    0.9864    0.3349    0.9228    0.9071    0.3214    1.0978    0.6376    0.2427    1.0042    1.3271    1.1992    1.4280    0.7404    0.1647    1.5829    1.6905    1.2277    1.2853    1.5601    1.4872    1.5287    1.4933    1.0119    0.9232    0.8388    0.5228    0.4626    0.9153    0.4370    0.3561    0.7138    0.9262    0.6735    0.4229    1.0059    1.3901    1.1567    1.1172    1.1663    0.8788    0.6201    0.5718    0.5504    0.7083    0.9754    0.8529    0.6212    1.0816    0.6189    0.8821    0.5947    1.0382    1.0419    1.0876    1.0255    0.8636    0.6648    0.5221    0.5914    0.5765    0.5728    0.3727    0.3779    0.5613    0.3542    0.3265    0.3302    0.3636    0.1811    0.2456    0.2864    0.2376    0.3892    0.2717    0.3302    0.2504    0.3295    0.4219    0.4752    0.2717    0.2456    0.2485    0.4911    0.4752    0.4242    0.5344    0.4654    0.9727    1.1560    1.2040    0.7301    0.7772    0.7205    0.8656    0.6335    0.4038    0.6784    0.9577    0.5184    0.3576    0.7858    0.7471    0.9485    0.6335    0.5491    0.3431    0.4315    0.3324    0.6126    0.2366    0.5128    0.5848    0.8960    0.9704    0.6766    1.0096    1.2860    1.2792    0.9383    0.6553    0.5975    0.9005    0.7225    0.6640    0.6834    0.6530    0.4911    0.4127    0.6091    0.3960    0.5312    0.4415    0.3954    0.3302    0.5600    0.5861    0.7410    0.7154    0.3622    0.6738];% Hand height, in mdata2=[    0.2540    0.1230    0.1770    0.2050    0.2750    0.2090    0.1110    0.2470    0.1440    0.2140    0.1190    0.1260    0.1560    0.1080    0.0980    0.0690    0.0540    0.1110    0.1400    0.0870    0.0920    0.2026    0.2723    0.1670    0.1183    0.1687    0.1170    0.1297    0.1232    0.1086    0.3449    0.1053    0.2253    0.2117    0.1896    0.1216    0.2093    0.1653    0.2464    0.1734    0.1054    0.1280    0.1750    0.1410    0.0891    0.1053    0.2026    0.1585    0.1379    0.1266    0.0786    0.1588    0.2107    0.2058    0.1621    0.1686    0.1880    0.1572    0.1475    0.1459    0.1232    0.1769    0.1848    0.1621    0.1304    0.1410    0.0908    0.1135    0.1118    0.1118    0.1929    0.1345    0.2081];% Hand speed while loading the stick, in m/sdata3=[    0.5993    0.9228    0.8801    0.3637    0.6839    0.0673    1.1314    0.7644    0.6609    0.4557    0.6913    1.1240    0.4392    0.6117    0.4655    0.7570    0.4499    0.0501    0.2151    0.1913    0.1125    0.6839    0.3966    0.1379    0.5608    0.3760    0.4557];
